# Maxillofacial Morphology as a Predictive Factor for Caries Risk in Orthodontic Patients: A Cross-Sectional Study

**DOI:** 10.3390/jcm13020622

**Published:** 2024-01-22

**Authors:** Yuma Koizumi, Ryo Kunimatsu, Isamu Kado, Yuki Yoshimi, Sakura Yamada, Tomohiro Ogasawara, Kotaro Tanimoto

**Affiliations:** 1 Department of Orthodontics, Division of Oral Health and Development, Hiroshima University Hospital, 1-2-3 Kasumi, Minami-ku, Hiroshima City 734-8554, Japan; ykoizumi@hiroshima-u.ac.jp (Y.K.); yukimihsoy@hiroshima-u.ac.jp (Y.Y.); syamada@hiroshima-u.ac.jp (S.Y.); t-ogasawara@hiroshima-u.ac.jp (T.O.); 2 Department of Orthodontics, Graduate School of Biochemical and Health Sciences, Hiroshima University, 1-2-3 Kasumi, Minami-ku, Hiroshima City 734-8554, Japan; isamu-kado@hiroshima-u.ac.jp (I.K.); tkotaro@hiroshima-u.ac.jp (K.T.)

**Keywords:** caries risk, orthodontics, maxillofacial morphology, salivary characteristics

## Abstract

This cross-sectional study aimed to explore the correlation between maxillofacial morphology and caries risk, assessed using salivary tests, in orthodontic patients. Despite enhancing the oral health-related quality of life, orthodontic treatment may adversely affect oral hygiene and increase caries risk. This study included 1071 patients all of whom underwent orthodontic examinations and salivary tests before starting orthodontic treatment at a hospital. Salivary tests were performed to assess the secretion rate, pH, buffering capacity, and counts of cariogenic bacteria. The maxillofacial morphology was evaluated using cephalometric X-rays and dental models. Statistical analyses revealed significant correlations among salivary characteristics, bacterial scores, and maxillofacial morphology. Notably, the facial angle and *Y*-axis values were associated with salivary secretion (*p* < 0.001), pH (*p* < 0.001), buffering capacity (*p* < 0.05), and cariogenic bacterial scores (*p* < 0.01), respectably. In conclusion, assessing the maxillofacial morphology before orthodontic treatment may aid in predicting the risk of bacterial oral diseases, offering valuable insights into personalized preventive measures. These findings underscore the potential for comprehensive evaluations to enhance caries risk assessment in orthodontic patients.

## 1. Introduction

Currently employed methods for assessing caries risk include a variety of approaches such as lifestyle history interviews; intraoral examinations; X-ray imaging; decayed, missing, and filled teeth (DMFT) index; Cariogram; and salivary tests [1,2,3,4,5]. Saliva serves as a representative defense factor against caries, contributing to caries prevention through various mechanisms, such as antimicrobial action, buffering capacity, tooth remineralization, and digestive function [6,7]. However, the amount of saliva secreted decreases with age [8]. Moreover, the amount and properties of salivary secretions differ according to sex [9,10,11]. A study comparing oral hygiene in men and women reported that although men could have poorer oral hygiene compared to women, they showed better saliva test results and lower *streptococcus mutans* (*S. mutans*) counts [11].

Orthodontic treatment could improve the oral health-related quality of life of patients, including aspects of mastication, occlusion, and esthetics [12,13]. However, using orthodontic appliances during orthodontic treatment, oral hygiene could deteriorate, resulting in changes in bacterial flora and an increased risk of bacterial diseases, such as white spot lesions, caries, and periodontitis [14,15,16,17,18]. Dental caries is irreversible once it causes substantial loss of tooth structure. Therefore, early detection and prevention of caries are crucial [19]. Based on this background, an accurate assessment of caries risk in individual patients before undergoing orthodontic treatment is essential [20]. Social factors, such as area of residence, occupation, and level of education, also influence caries risk. Of particular importance, however, are individual-level factors, such as the content and frequency of meals, oral hygiene, fluoride use, and saliva function [21]. Evaluating the quantity and quality of patient saliva before starting orthodontic treatment is highly valuable for assessing the risk of caries during orthodontic treatment [22,23]. Saliva-based caries risk assessment has gained attention as a non-invasive and convenient method for evaluating the patients’ oral condition; many dental clinics perform caries risk assessments using saliva. However, reports addressing the impact of maxillofacial morphology on salivary characteristics are scarce, although malocclusion has been reported to increase the risk of caries [24,25]. Furthermore, the correlation between maxillofacial morphology and caries risk remains unclear.

Therefore, this study aimed to investigate the correlation between the results of salivary tests and maxillofacial morphology. Moreover, this study sought to verify the feasibility of predicting caries risk in patients using salivary characteristics and counts of cariogenic bacteria.

## 2. Materials and Methods

### 2.1. Study Design and Ethical Statement

This cross-sectional study was approved by the appropriate Ethics Review Board (E-1039). The study participants were retrospectively selected using clinical records that met all the necessary items for the study. Since patients under 18 years of age were included, consent was obtained from their legal guardians.

### 2.2. Study Population

A total of 1071 patients (401 male and 670 female patients, age range: 4–71 years, mean age: 14.8 ± 9.8 years) who visited the orthodontic department of one hospital for initial examinations, salivary tests (caries risk assessment), and plaque control record (PCR) between April 2015 and March 2023 were included. All patients agreed to participate in the study. The salivary test included assessments of stimulated saliva secretion, pH, buffering capacity, and counts of *S. mutans* and *Lactobacillus* species. Maxillofacial morphology and occlusal status were evaluated using lateral cephalometric radiographs and dental models.

### 2.3. Saliva Collection and Oral Examination

The patients were instructed to avoid vigorous exercise, eating, smoking, and tooth brushing at least 2 h before the saliva test and avoid using antiseptic mouthwash within 6 h before saliva testing. The patients were asked to chew tasteless paraffin gum for 1 min while seated on a dental chair. After removing the gum, for the caries risk test (CRT) (Ivoclar Vivadent, Tokyo, Japan) or Dentocult (OralCare, Tokyo, Japan), the test surface of the strip mutans was rubbed on the patient’s tongue surface for 5 back-and-forth motions. The strip was then placed inside the cap of a test tube containing bacitracin tablets inserted 15 min earlier. Subsequently, the same paraffin gum was chewed for 5 min, and the stimulated saliva was collected in a conical tube. If a sufficient amount of saliva could not be collected, the patient was asked to chew the gum for additional few minutes. The collected saliva and bacteria were immediately tested. PCR was recorded by the dental hygienist at the time of initial examination.

### 2.4. Saliva Testing

The saliva secretion rate (mL/min) was calculated based on the total amount of collected saliva. The saliva pH was measured using a Checkbuf pH meter (Horiba, Kyoto, Japan). The buffering capacity of saliva was evaluated using the CAT21Buf risk test (Morita Co., Osaka, Japan) [26]. *S. mutans* and *Lactobacillus* counts were measured using CRT caries risk test or Dentocult, cultured at 37 °C for 48 h, and evaluated on a 4-point scale for each test, calculating the bacterial score (for CRT caries risk test: score 1, 2: <10^5^ colony forming unit [CFU], score 3, 4: >10^5^ CFU; for Dentocult: score 1: <10^5^ CFU, score 2: <10^6^ CFU, score 3: 10^6^–10^7^ CFU, score 4: >10^7^ CFU). The CRT caries risk test was used for patients presenting between April 2015 and March 2020, and the Dentocult was used for patients presenting between April 2020 and March 2023.

### 2.5. Maxillofacial Morphological Analysis

Lateral cephalometric radiographs were traced, and the traced data were analyzed using analysis software (COA5 version 1.8.1; JM Ortho, Tokyo, Japan). The anatomical landmarks, points, and angles used in this study are shown in Table 1. Linear measurements of overjet (OJ) and overbite (OB) were performed using dental models and calipers. All measurements were performed by the orthodontists at a hospital.

### 2.6. Statistical Analysis

Multiple regression analysis was used to evaluate the correlation between maxillofacial morphology and the results of the caries risk assessment tests and PCR. The *p* value < 0.05 was set as the significant value for partial regression coefficients. The confidence interval of the population mean was calculated using a 95% confidence level. BellCurve for Excel (Social Survey Research Information Co., Ltd., Tokyo, Japan) was used to evaluate correlations and calculate confidence intervals. After performing the test, we calculated the test power using G*Power 3.1 software (Heinrich Heine University, Düsseldorf, Germany) to check the degree of statistical power.

## 3. Results

### 3.1. Statistical Analysis Results of Each Parameter

Table 2 shows the average values, standard deviations, and confidence intervals of the anatomical landmarks, points, angles, OJ, and OB used in the correlation analysis. Table 3 shows the average value, standard deviation, and statistical power of the five items investigated in the caries risk test and PCR. The statistical power for saliva flow rate, salivary pH, and *S. mutans* score was high; however, the statistical power for salivary buffering capacity, *Lactobacillus* score, and PCR was below 0.8.

### 3.2. Correlation between Salivary Secretion Amount, Characteristics, and Maxillofacial Morphology

**Figure 1:**
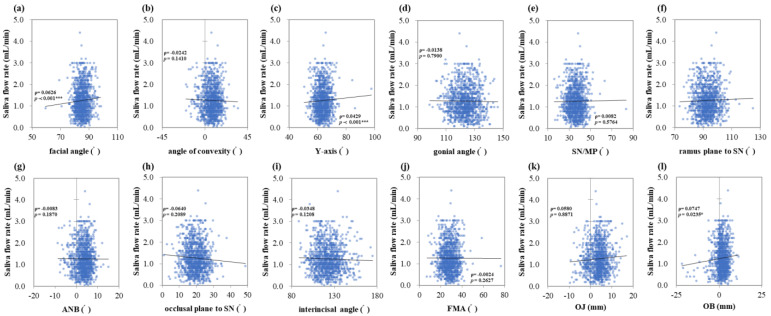
Correlations between the saliva flow rate and maxillofacial morphology. Multiple regression analysis was used to determine the correlations between (**a**) the facial angle, (**b**) angle of convexity, (**c**) *Y*-axis, (**d**) gonial angle, (**e**) SN/MP, (**f**) ramus plane to SN, (**g**) ANB, (**h**) occlusal plane to SN, (**i**) interincisal angle, (**j**) FMA, (**k**) OJ, and (**l**) OB. Each subject is indicated by a dot, and linear regression is indicated by a straight line. Single correlations are indicated by ρ and significant differences by *p* < 0.05 *, *p* < 0.001 ***, respectively.

**Figure 2:**
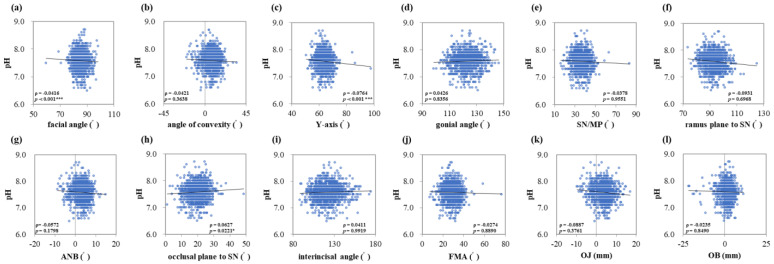
Correlations between salivary pH and maxillofacial morphology. Multiple regression analysis was used to determine the correlations between (**a**) the facial angle, (**b**) angle of convexity, (**c**) *Y*-axis, (**d**) gonial angle, (**e**) SN/MP, (**f**) ramus plane to SN, (**g**) ANB, (**h**) occlusal plane to SN, (**i**) interincisal angle, (**j**) FMA, (**k**) OJ, and (**l**) OB. Each subject is indicated by a dot, and linear regression is indicated by a straight line. Single correlations are indicated by ρ and significant differences by *p* < 0.05 *, *p* < 0.001 ***, respectively.

**Figure 3:**
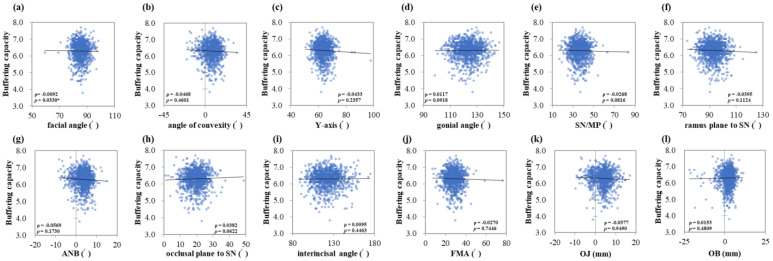
Correlations between the salivary buffering capacity and maxillofacial morphology. Multiple regression analysis was used to determine the correlations between (**a**) the facial angle, (**b**) angle of convexity, (**c**) *Y*-axis, (**d**) gonial angle, (**e**) SN/MP, (**f**) ramus plane to SN, (**g**) ANB, (**h**) occlusal plane to SN, (**i**) interincisal angle, (**j**) FMA, (**k**) OJ, and (**l**) OB. Each subject is indicated by a dot, and linear regression is indicated by a straight line. Single correlations are indicated by ρ and significant differences by *p* < 0.05 *.

The saliva flow rate showed positive correlations with the facial angle (ρ = 0.0626, *p* < 0.001 ***), *Y*-axis (ρ = 0.0429, *p* < 0.001 ***), and OB (ρ = 0.0747, *p* = 0.0235 *) (Figure 1a,c,l). Salivary pH showed negative correlations with the facial angle (ρ = −0.0416, *p* < 0.001 ***), *Y*-axis (ρ = −0.0764, *p* < 0.001 ***) and positive correlation with occlusal plane to SN (ρ = 0.0627, *p* = 0.0221 *) (Figure 2a,c,h). Salivary buffering capacity showed a negative correlation with the facial angle (ρ = −0.0092, *p* = 0.0330 *) (Figure 3a). As the facial angle increased, the saliva-flow rate also increased, whereas pH and buffering capacity decreased. Similarly, the salivary flow rate showed a positive correlation with the *Y*-axis; however, salivary pH showed a negative correlation with the *Y*-axis. The angle of convexity, gonial angle, SN/MP, ramus plane to the SN, ANB, interincisal angle, FMA, and OJ showed no correlation with the amount of salivary secretion or other characteristics.

### 3.3. Correlation between Cariogenic Bacterial Score and Maxillofacial Morphology

**Figure 4:**
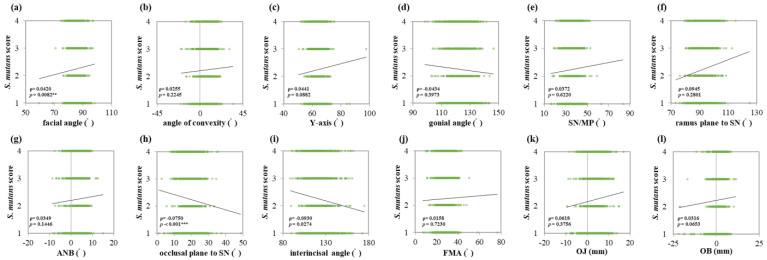
Correlations between *S. mutans* score and maxillofacial morphology. Multiple regression analysis was used to determine the correlations between (**a**) the facial angle, (**b**) angle of convexity, (**c**) *Y*-axis, (**d**) gonial angle, (**e**) SN/MP, (**f**) ramus plane to SN, (**g**) ANB, (**h**) occlusal plane to SN, (**i**) interincisal angle, (**j**) FMA, (**k**) OJ, and (**l**) OB. Each subject is indicated by a dot, and linear regression is indicated by a straight line. Single correlations are indicated by ρ and significant differences by *p* < 0.01 **, and *p* < 0.001 ***, respectively.

**Figure 5:**
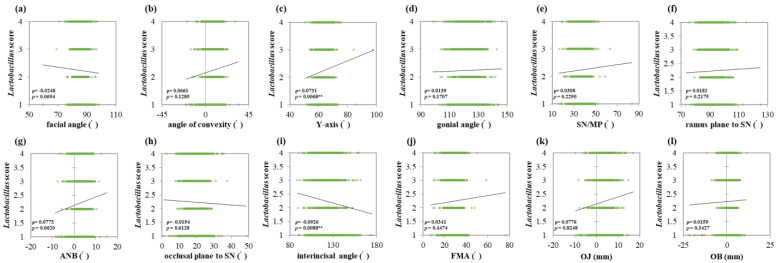
Correlations between *Lactobacillus* score and maxillofacial morphology. Multiple regression analysis was used to determine the correlations between (**a**) the facial angle, (**b**) angle of convexity, (**c**) *Y*-axis, (**d**) gonial angle, (**e**) SN/MP, (**f**) ramus plane to SN, (**g**) ANB, (**h**) occlusal plane to SN, (**i**) interincisal angle, (**j**) FMA, (**k**) OJ, and (**l**) OB. Each subject is indicated by a dot, and linear regression is indicated by a straight line. Single correlations are indicated by ρ and significant differences by *p* < 0.01 **.

*S. mutans* score showed a strong positive correlation with the facial angle (ρ = 0.0420, *p* = 0.0082 **) (Figure 4a). Conversely, the occlusal plane to SN (ρ = −0.0750, *p* < 0.001 ***) and interincisal angle (ρ = −0.0930, *p* = 0.0274 *) showed negative correlations (Figure 4h,i). *Lactobacillus* score showed a positive correlation with the *Y*-axis (ρ = 0.0731, *p* = 0.0068 **) and negative correlation with the interincisal angle (ρ = −0.0926, *p* = 0.0088 **) (Figure 5c,i). The interincisal angle showed negative correlations with both *S. mutans* and *Lactobacillus* scores.

### 3.4. Correlation between PCR and Maxillofacial Morphology

**Figure 6:**
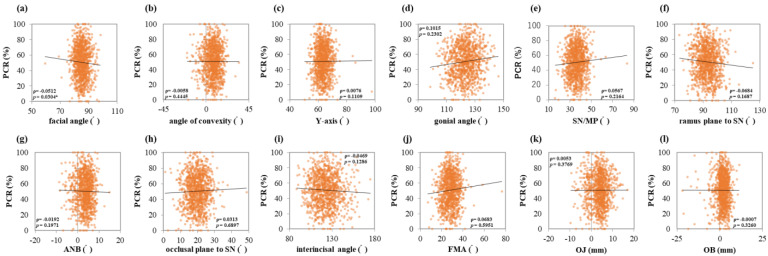
Correlations between PCR and maxillofacial morphology. Multiple regression analysis was used to determine the correlation between (**a**) the facial angle, (**b**) angle of convexity, (**c**) *Y*-axis, (**d**) gonial angle, (**e**) SN/MP, (**f**) ramus plane to SN, (**g**) ANB, (**h**) occlusal plane to SN, (**i**) interincisal angle, (**j**) FMA, (**k**) OJ, and (**l**) OB. Each subject is indicated by a dot, and linear regression is indicated by a straight line. Single correlations are indicated by ρ and significant differences by *p* < 0.05 *.

PCR showed a negative correlation with the facial angle (ρ = −0.0512, *p* = 0.0304 *) (Figure 6a). No other angles were correlated with PCR.

## 4. Discussion

This study investigated the correlations among salivary tests, cariogenic bacteria, and maxillofacial morphology. The results of this study revealed a close association between the facial angle and salivary characteristics, including the *S. mutans* score. Particularly, the saliva-flow rate showed a positive correlation with the facial angle and the *Y*-axis. This may be due to an increase in the individual’s gum chewing efficiency with an increase in their facial angle and *Y*-axis, thereby increasing stimulated saliva secretion. Moreover, salivary pH and salivary buffering capacity showed a negative correlation with the facial angle and *Y*-axis. Therefore, the results of this study suggest that salivary protein concentrations may differ according to specific maxillofacial morphology. However, further studies are needed to determine how the facial angle and *Y*-axis values are related to caries risk. Additionally, Preethi et al. reported that the flow rate, pH, and buffering capacity were slightly decreased in the saliva of children affected by caries [27]. Since this study focused on the correlation between caries risk testing and maxillofacial morphology, the inclusion of a patient’s oral status, such as the presence of caries, as an investigation item may be further explored. No correlation was found between the saliva-flow rate, salivary properties, and angle of convexity. Moreover, no correlation was observed between the anteroposterior position of the maxilla and salivary properties in this study.

*S. mutans* score positively correlated with the facial angle, whereas the *Lactobacillus* score showed a positive correlation with the *Y*-axis. *S. mutans* has a strong ability to form biofilms and is a typical caries-causing bacterium [28,29]. The facial angle and saliva-flow rate are positively correlated and known to inhibit the growth of *S. mutans*. Therefore, other factors may be responsible for this result. The risk of dental caries is higher with the intake of carbohydrate-rich diets, particularly with frequent consumption [30,31]. These factors may have contributed to our results. In contrast, *Lactobacillus* species in the oral cavity cause a decline in oral pH by fermenting sugars and promoting biofilm formation and are strongly associated with the development of dentin caries [32]. Thus, in patients with a large *Y*-axis, the results indicated an increased risk of dental caries. Both the facial angle and *Y*-axis showed positive correlations with the saliva-flow rate and cariogenic bacterial scores. Therefore, how these two factors could affect total caries risk assessment remains unclear. The interincisal angle negatively correlated with both *S. mutans* and *Lactobacillus* scores. A small angle of inclination of the upper and lower anterior teeth axis is considered to suggest difficulty in cutting food with the front teeth during meals, resulting in a prolonged mealtime and, consequently, a decline in oral pH and subsequent bacterial growth in the oral cavity.

The PCR values showed a negative correlation with the facial angle, indicating that patients with a skeletal tendency toward mandibular protrusion have a higher level of oral hygiene than patients with a skeletal tendency toward maxillary protrusion. At this stage, it is not possible to determine the cause of this result. As previously reported, oral hygiene is poor when there is crowding; hence, a small facial angle may indicate that the alveolar bone itself is small, and crowding is observed [33]. Therefore, it is desirable to evaluate the amount of crowding in the future.

A limitation of this study is that it only investigated the correlation between caries risk assessment results and maxillofacial morphology. Other examination items such as age, sex, eating habits, contents of meals, presence of carious teeth, underlying diseases, and social factors would have provided more detailed insights. Moreover, the inclusion of all patients with available data is a limitation because the optimal sample size of the study population is unclear.

Future studies should investigate the relationship between maxillofacial morphology and caries prevalence in patients undergoing orthodontic treatment. Combining the results with those of the CRT could enable the assessment of each item in the caries risk evaluation test, identifying maxillofacial morphologies at a heightened risk of caries. Further research should be conducted to develop a more accurate screening test for assessing caries risk before orthodontic treatment.

## 5. Conclusions

The saliva-flow rate showed a strong positive correlation with the facial angle and the *Y*-axis. Salivary pH negatively correlated with the facial angle and *Y*-axis. Salivary buffering capacity negatively correlated with the facial angle. Both cariogenic bacterial scores negatively correlated with the interincisal angle. *S. mutans* score positively correlated with the facial angle, and the *Lactobacillus* score positively correlated with the *Y*-axis. The evaluation of specific maxillofacial morphologies showed a correlation with the assessment of salivary characteristics and cariogenic bacterial counts.

## Figures and Tables

**Table 1:** Anatomical landmarks, points, and angles.

Anatomical Indicators	Definition
Landmarks and points	
S (sella)	Center of sella turcica
N (nasion)	The most anterior point of the frontonasal suture
Or (orbitale)	The lowest point on the average left and right inferior borders of the bony orbit
Po (porion)	The highest point on the superior surface of the soft tissue of the external auditory meatus
A-Point	The most posterior point on the anterior contour of the upper alveolar process
B-Point	The most posterior point on the anterior contour of the lower alveolar process
Me (menton)	The lowest point on the mandibular symphysis
Pog (Pogonion)	The most anterior point of the midline cross-sectional image of the mandibular miter
Gn (gnathion)	The midpoint of the nasolabial groove
Ar (articulare)	The intersection of the posterior margin of the mandibular branch and subnasal margin of the occipital bone
U1	The tip of the maxillary central incisor
L1	The tip of the mandibular central incisor
Mo	The central point of the cuspid fit of the upper and lower first molars
Angles	
Facial angle (°)	The angle between the Frankfurt (FH; Or-Po) plane and N-Pog plane
Angle of convexity (°)	The angle between the straight-line NA and A-Pog plane
*Y*-axis (°)	The angle between the S-Gn and FH plane
Gonial angle (°)	The angle between the ramus plane (a tangent line between the Ar and posterior margin of the mandibular branch) and mandibular plane
SN/MP (°)	The angle between the SN plane and the mandibular plane
Ramus plane to SN (°)	The angle between the ramus plane and SN plane
ANB (°)	The angle formed by point A, nasion, and point B
Occlusal plane to SN (°)	The angle between the line connecting Mo and midpoints of U1 and L1 and the SN plane
FMA (°)	The angle the between mandibular inferior margin plane and the FH plane
Interincisal angle (°)	The angle between the long axis of the U1 and the long axis of the L1

**Table 2:** The confidence intervals for each parameter.

Variables			95% Confidence Level	
	Average	S.D.	Lower Bound	Upper Bound
Facial angle (°)	85.254	3.846	85.023	85.485
Angle of convexity (°)	7.151	6.955	6.734	7.569
*Y*-axis (°)	63.423	4.059	63.180	63.667
Gonial angle (°)	123.164	7.289	122.727	123.601
SN/MP (°)	36.151	6.240	35.776	36.525
Ramus plane to SN (°)	92.979	5.908	92.625	93.334
ANB (°)	3.329	3.081	3.144	3.514
Occlusal plane to SN (°)	19.695	4.899	19.401	19.989
Interincisal angle (°)	122.612	12.861	121.840	123.384
FMA (°)	28.199	6.118	27.832	28.566
OJ (mm)	3.669	3.701	3.447	3.891
OB (mm)	1.678	3.268	1.482	1.874

Abbreviations: OJ, overjet; OB, overbite.

**Table 3:** The statistical powers of each parameter.

Variables	Average	S.D.	Statistical Power
Saliva			
Saliva flow rate (mL/min)	1.262	0.671	0.9918855
pH	7.578	0.322	0.9997623
Buffering capacity	6.303	0.521	0.5242072
Bacterial score			
*S. mutans* score	2.241	1.234	0.9988603
*Lactobacillus* score	2.239	1.222	0.7908876
PCR (%)	50.659	20.992	0.7182778

Abbreviation: PCR, plaque control record, S.D., standard deviation.

## Data Availability

The data supporting the findings of this study are available from the corresponding author upon reasonable request.
